# Serine-Threonine Kinase Receptor-Associated Protein (STRAP) Knockout Decreases the Malignant Phenotype in Neuroblastoma Cell Lines

**DOI:** 10.3390/cancers13133201

**Published:** 2021-06-26

**Authors:** Laura V Bownes, Adele P Williams, Raoud Marayati, Colin H Quinn, Sara C Hutchins, Jerry E Stewart, Trung Vu, Juliet L Easlick, Elizabeth Mroczek-Musulman, David K Crossman, Joshua C Anderson, Christopher D Willey, Pran K Datta, Elizabeth A Beierle

**Affiliations:** 1 Division of Pediatric Surgery, Department of Surgery, University of Alabama at Birmingham, Birmingham, AL 35233, USA; lbownes@uabmc.edu (L.V.B.); awil26@lsuhsc.edu (A.P.W.); rmarayati@uabmc.edu (R.M.); chquinn@uab.edu (C.H.Q.); schutchins@uabmc.edu (S.C.H.); jessy@uab.edu (J.E.S.); 2 Division of Hematology and Oncology, Department of Medicine, University of Alabama at Birmingham, Birmingham, AL 35233, USA; ttvu@uab.edu; 3 Division of Transplantation, Department of Surgery, University of Alabama at Birmingham, Birmingham, AL 35233, USA; julieteaslick@uabmc.edu; 4 Department of Pathology, Children’s of Alabama, Birmingham, AL 35233, USA; Elizabeth.Mroczek-Musulman@childrensal.org; 5 Department of Genetics, University of Alabama at Birmingham, Birmingham, AL 35233, USA; dkcrossm@uab.edu; 6 Department of Radiation Oncology, University of Alabama at Birmingham, Birmingham, AL 35233, USA; janders7@uab.edu (J.C.A.); cwilley@uab.edu (C.D.W.)

**Keywords:** serine-threonine kinase receptor-associated protein, neuroblastoma, CRISPR-Cas9

## Abstract

**Simple Summary:**

Neuroblastoma is the most common extra-cranial tumor in children and despite medical advancements in cancer treatment, five-year survival for high-risk neuroblastoma remains less than 50%. Investigation of the mechanisms responsible for aggressive disease is necessary to identify novel therapeutic targets and improve survival. Serine-threonine kinase receptor associated protein (STRAP) is upregulated in several malignancies and plays an important role in tumor growth and metastasis. The role of STRAP in pediatric malignancies and specifically in neuroblastoma has not been explored. We sought to determine whether STRAP functions assisted to promote the malignant phenotype in neuroblastoma and could provide a potential target for future therapies.

**Abstract:**

**Background:** Serine-threonine kinase receptor-associated protein (STRAP) plays an important role in neural development but also in tumor growth. Neuroblastoma, a tumor of neural crest origin, is the most common extracranial solid malignancy of childhood and it continues to carry a poor prognosis. The recent discovery of the role of STRAP in another pediatric solid tumor, osteosarcoma, and the known function of STRAP in neural development, led us to investigate the role of STRAP in neuroblastoma tumorigenesis. **Methods:** STRAP protein expression was abrogated in two human neuroblastoma cell lines, SK-N-AS and SK-N-BE(2), using transient knockdown with siRNA, stable knockdown with shRNA lentiviral transfection, and CRISPR-Cas9 genetic knockout. STRAP knockdown and knockout cells were examined for phenotypic alterations in vitro and tumor growth in vivo. **Results:** Cell proliferation, motility, and growth were significantly decreased in STRAP knockout compared to wild-type cells. Indicators of stemness, including mRNA abundance of common stem cell markers Oct4, Nanog, and Nestin, the percentage of cells expressing CD133 on their surface, and the ability to form tumorspheres were significantly decreased in the STRAP KO cells. In vivo, STRAP knockout cells formed tumors less readily than wild-type tumor cells. **Conclusion:** These novel findings demonstrated that STRAP plays a role in tumorigenesis and maintenance of neuroblastoma stemness.

## 1. Introduction

Despite advancements in pediatric cancer care, neuroblastoma, a childhood malignancy resulting from abnormal neural crest cell development, remains the cause of over 15% of pediatric cancer related deaths [1]. Current treatment regimens for high-risk disease include chemotherapy, surgical resection, autologous stem cell transplant, radiation, immunotherapy, and retinoic acid [2]. Despite these extensive therapies, outcomes remain dismal for patients with high-risk disease as less than 50% will attain a 5 year survival [3]. It is crucial to continue to investigate this disease in order to develop new therapies, especially for the cohort of patients with high-risk disease.

Serine-threonine kinase receptor-associated protein (STRAP) is a scaffolding protein that facilitates protein to protein interactions [4]. Investigators began to study the role of STRAP in cancer when they found that STRAP inhibited the tumor suppressor, TGF-β [5]. STRAP is overexpressed in several malignancies, including colorectal [6] and lung cancer [7] and the pediatric bone cancer, osteosarcoma [8]. In these malignancies, STRAP enhanced cancer cell proliferation and tumor growth.

STRAP expression supports tumorigenicity by promoting the Wnt/β-catenin pathway in colorectal cancer. In cells overexpressing STRAP, silencing the STRAP gene with short hairpin RNAs (shRNA) resulted in decreased invasion and metastasis [6]. In lung cancer, STRAP led to tumor progression by downregulating tumor suppressors, E-cadherin, and the CDK inhibitor, p21Cip1, through the modulation of the transcription factor Sp1 [9]. Pruksakorn et al. found STRAP was upregulated in osteosarcoma and inhibiting STRAP with small interfering RNA (siRNA) decreased migration and invasion; this suggests that STRAP contributed to osteosarcoma metastasis [8]. These discoveries lend support for investigating STRAP in neuroblastoma. We hypothesized that inhibition of STRAP protein expression in neuroblastoma would result in a decrease in the malignant phenotype in vitro and in vivo. In the current study, we demonstrate STRAP inhibition with siRNA, shRNA, and CRISPR-Cas9 knockout (KO) decreased tumor cell viability, proliferation, growth, stemness, and motility in vitro and tumor growth in vivo. To our knowledge, researchers have not performed extensive study of STRAP in neuroblastoma and our findings provide evidence for STRAP as a potential driver for neuroblastoma tumorigenesis.

## 2. Materials and Methods

### 2.1. Cells and Cell Culture

The human neuroblastoma cell lines SK-N-AS (AS, CRL-2137, and MYCN non-amplified), SK-N-BE (2) (BE, CRL-2271, and MYCN amplified), and SH-SY5Y (CRL-2266 and MYCN non-amplified) were obtained from the American Type Culture Collection (ATCC, Manassas, VA, USA). The isogenic SH-EP (SHEP) and WAC (2) (WAC), MYCN non-amplified and amplified cell lines, respectively, were a gift from M. Schwab (Deutsches Krebsforschungszentrum, Heidelberg, Germany) [10]. Cells were maintained under standard culture conditions at 37 °C and 5% CO_2_. AS cells were maintained in Dulbecco’s modified Eagle’s medium (DMEM, 30-2601, ATCC) containing 10% fetal bovine serum (FBS, Hyclone, Suwanee, GA, USA), 4 mM L-glutamine (Thermo Fisher Scientific Inc., Waltham, MA, USA), 1 µM non-essential amino acids, and 1 µg/mL penicillin/streptomycin (Gibco, Carlsbad, CA, USA), which is referred to as media. BE cells were maintained in a 1:1 mixture of minimum Eagle medium and Ham F-12 medium (30–2004, ATCC) with 10% FBS (Hyclone), 1 μM non-essential amino acids, 2 mM l-glutamine (Thermo Fisher Scientific Inc.), and 1/μg/mL penicillin/streptomycin (Gibco). SH-EP and WAC (2) were maintained in Roswell Park Memorial Institute (RPMI) 1640 medium (30–2001, ATCC) with 10% FBS (Hyclone), 2/mM l-glutamine (Thermo Fisher Scientific Inc.), and 1 μg/mL penicillin/streptomycin (Gibco). All cell lines were verified within the last 12 months using short tandem repeat analysis (University of Alabama at Birmingham (UAB) Genomics Core, Birmingham, AL, USA) and tested for and deemed free of mycoplasma infection.

### 2.2. Small Interfering RNA (siRNA) Inhibition of STRAP

AS or BE cells (1 × 10^6^) were plated in 6-well plates and transfected for 72 h with Lipofectamine RNAiMax (Thermo Fisher Scientific) or siRNA directed to either control (siNeg), siSTRAP1, siSTRAP2, or a combination of the two STRAP siRNAs at 20 µM concentration with Lipofectamine RNAiMax (Thermo Fisher Scientific). SiNeg (ON-TARGETplus Non-targeting siRNA #1) was obtained from Dharmacon (GE Dharmacon, Thermo Fisher Scientific, Lafayette, CO, USA). The siSTRAP1 (SASI_Hs01_00016957) and siSTRAP2 (SASI_Hs02_00343131) were obtained from Sigma Aldrich (St. Louis, MO, USA).

### 2.3. Short Hairpin RNA (shRNA) Inhibition of STRAP

The shScramble and shSTRAP cell lines were generously provided by Dr. Pran Datta’s laboratory and were established as previously described [11]. The shScramble served as the control for shSTRAP cells. AS cells that underwent lentiviral transfection with shScramble or shSTRAP were cultured in AS media with the addition of puromycin (5 µM, P8833, Sigma-Aldrich, St. Louis, MO, USA) for selection.

### 2.4. CRISPR-Cas9 Knockout (KO) of STRAP

The CRISPR vector, pSpCas9(BB)-2A-GFP (pX458), was a gift from Dr. A. Joseph Tector and developed by Dr. Feng Zhang (Addgene plasmid #48138) [12]. We used Geneious software (Biomatters, Auckland, New Zealand) to design guide RNAs (gRNAs) to regions from the 5′ untranslated region through exon 3 of the STRAP gene. The selected gRNAs were evaluated with the MIT CRISPR Design Tool (http://crispr.mit.edu/) to assess for potential off-target sequences. The oligonucleotides (5′-CACCGTTGGGGTGCAACACTGAATA-3′ and 5′-CAACCCCACGTTGTGACTTATCAAAA-3′) were annealed at 37 °C for 30 min, 95 °C for 5 min, and ramped down to 25 °C at 5 °C per minute. Annealed oligonucleotides were cloned into the CRISPR plasmid by digesting 1 µg of plasmid pX458 with BbsI (New England Biolabs, Ipswich, MA, USA) in the presence of annealed oligonucleotides, T7 ligase, and ATP in a MyCycler™ thermal cycler (Bio-Rad, Hercules, CA, USA) for 37 °C for 5 min and 23 °C for 5 min for 6 cycles. Ligation reaction was transformed into Invitrogen MAX Efficiency™ DH5α™ competent *E. coli* cells (Invitrogen) following the manufacturer’s protocol. Plasmids were isolated from one colony per treatment using the QIAprep^®^ Miniprep (Qiagen, Germantown, MD, USA). DNA was isolated using QIAprep Spin Miniprep Kit (Qiagen) and sequenced by the UAB Genomics Core. AS or BE cells (2 × 10^6^) were plated in 6-well plates and 24 h later, transfection was carried out using FuGENE^®^ HD Transfection Reagent (Promega, Madison, WI, USA) per the manufacturer’s protocol. The STRAP gRNA plasmid was incubated for 15 min at room temperature in OptiMEM™ media (Thermo Fisher Scientific) with FuGENE^®^ HD Transfection Reagent (Promega) in 3:2 ratio of the transfection reagent to DNA. Plasmid DNA (10 µg) was added to the cells while swirling the flask. Forty-eight hours after transfection, cells from the plasmid transfection were sorted based on green fluorescent protein (GFP) expression using a FACSAria II cell sorter (BD Biosciences, San Jose, CA, USA) into 96-well plates with a single cell per well (Comprehensive Flow Cytometry Core, UAB, Birmingham, AL, USA). In order to screen for CRISPR-Cas9 mediated deletions of the STRAP gene, genomic DNA was isolated using the DNeasy Blood & Tissue Kit (Qiagen) from AS or BE wild-type (WT) cells and those clones that had grown to confluence and survived passage into larger flasks. Pwo SuperYield DNA Polymerase, dNTPack (Sigma Aldrich) with GC-rich solution were utilized per manufacturer’s protocol to amplify the region of interest within the STRAP gene using the following primers, (forward: 5′-TTAGTGCCTTCAGTGGGTGG-3′, reverse: 5′-GGTGGGATCAAACATGCGTTC-3′), which were designed using Primer-Blast (https://www.ncbi.nlm.nih.gov/tools/primer-blast/ (accessed on 3 September 2018)). PCR products were assessed using gel electrophoresis on a 1% agarose gel. Individual bands were cut and DNA was purified using the QIAquick Gel Extraction Kit (Qiagen). Nucleotide sequences of these DNA fragments were analyzed by Sanger sequencing (UAB Genomics Core) and aligned to the human reference sequence using the basic local alignment search tool (BLAST, NCBI, https://pubmed.ncbi.nlm.nih.gov/22708584/ (accessed on 27 February 2019)). STRAP protein expression in the AS or BE WT cells and selected STRAP KO clones was assessed by Western blotting to confirm the absence of the STRAP protein.

### 2.5. Rescue of STRAP Expression

In order to validate that the phenotypic changes noted with STRAP KO cells were not secondary to off-target effects of the CRISPR-Cas9 system, we performed rescue experiments by transfecting AS STRAP KO cells with c-Flag pc-DNA empty vector (EV) or pc-DNA STRAP plasmid [13]. STRAP KO cells (3 × 10^3^) were plated in 6-well plates and transfected with either FuGENE^®^ HD, FuGENE^®^ HD and EV plasmid, or FuGENE^®^ HD and STRAP plasmid for 72 h. Stable rescue cells were selected and maintained in AS media with the addition of G418 (600 µg/mL, A1720, Sigma-Aldrich). Cells were used in proliferation studies with CellTiter 96^®^ assay as described below to examine the phenotype of the KO cells following the re-introduction of STRAP.

### 2.6. Reagents and Antibodies

Trypan blue stain was obtained from Life Technologies Corporation (Grand Island, NY, USA). Primary antibodies used for Western blotting included the following: polyclonal rabbit anti-STRAP (18277-1-AP) from Proteintech (Rosemont, IL, USA), monoclonal rabbit anti-PGDFRβ (#3169) and monoclonal rabbit anti-vinculin (E1E9V, #13901) from Cell Signaling (Danvers, MA, USA), and monoclonal mouse anti-FLAG (F3165) and monoclonal mouse anti-β-actin (A1978) from Sigma Aldrich. Antibodies were used according to manufacturers’ suggestions.

### 2.7. Immunoblotting

Cells or homogenized tumor specimens were lysed on ice in a buffer consisting of 50 mM Tris-HCl (pH 7.4), 150 mM NaCl, 1 mM EDTA, 1% Triton x-100, 1% sodium deoxcycholate, 0.1% SDS, phosphatase inhibitor (P5726, Sigma Aldrich), protease inhibitor (P8340, Sigma Aldrich), and phenylmethylsulfonyl fluoride (PMSF, P7626, Sigma Aldrich) for 30 min. Lysates were centrifuged at 17,000× *g* for 30 min at 4 °C. Protein concentrations were determined using a Micro BCA™ Protein Assay Kit (Thermo Fisher Scientific). Proteins were separated on SDS-PAGE gels by electrophoresis and transferred to Immobilon^®^-P polyvinylidene fluoride (PVDF) transfer membrane (EMD Millipore, Burlington, MA, USA). In order to confirm the expected size of target proteins, Precision Plus Protein Kaleidoscope Standards (161-0375, Bio-Rad) molecular weight markers were used. Antibodies were used per the manufacturers’ recommended protocol. Luminata Classico or Luminata Crescendo (EMD Millipore) substrates were used to visualize immunoblots by enhanced chemiluminescence (ECL) of horseradish peroxidase (HPR)-conjugated secondary antibodies. β-actin or vinculin served as a control to ensure equal protein loading. We performed densitometry of Western blots using ImageJ software (Ver 1.49, http://imagej.nih.gov/ij (accessed on 7 July 2018).

### 2.8. Proliferation

Proliferation was examined using the CellTiter 96^®^ Aqueous One Solution Cell Proliferation assay (Promega). Cells (5 × 10^3^ cells) were plated onto 96-well plates. After 24 h, CellTiter 96^®^ dye (10 µL) was added to each well and the absorbance was measured at 490 nm using a microplate reader (Epoch Microplate Spectrophotometer, BioTek Instruments, Winooski, VT, USA). For the siRNA experiments, we transfected the cells with siRNA for 72 h as described above, then we lifted and plated transfected cells onto 96-well plates for 24 h, added CellTiter96^®^ dye (Promega), and read the plates. Proliferation experiments were completed with at least three biologic replicates and data reported as fold change ± standard error of the mean (SEM).

### 2.9. Growth Curve

Cells (5 × 10^4^) were plated in a 12-well plate in adherent conditions in 12-well plates. In order to measure cell growth over time, cells were lifted and live cells were counted after being stained with trypan blue at 1, 2, 3, and 4 days for AS cells and at 5, 7, and 9 days for BE cells.

### 2.10. Cell Cycle

AS WT and STRAP KO cells were plated in 6-well plates and maintained in AS media with decreased FBS (4%). After 24 h, cells (5 × 10^5^) were washed with PBS and fixed with 100% ethanol at 4 °C for at least 30 min. After a second wash with PBS, cells were stained with propidium iodide (Invitrogen), 0.1% TritonX (Active Motif, Carlsbad, CA, USA), and RNAse A (0.1 mg/mL, Qiagen) and cell cycle data were obtained using the FACSCalibur™ Flow Cytometer (BD Biosciences) and analyzed using the FlowJo software (FlowJo, LLC, Ashland, OR, USA).

### 2.11. Migration and Invasion

The effect of STRAP KO or shRNA inhibition on migration was assessed using modified Boyden chamber assays. Cells (4 × 10^4^) were seeded onto 8 µM pore inserts (TransWell^®^, Corning, Corning, NY, USA) and were allowed to migrate for 24 h with laminin (10 μg/mL, 100 μL, Trevigen, Gaithersburg, MD, USA) used in the outer well as a chemoattractant. Inserts were fixed with 4% paraformaldehyde for 10 min, stained with 1% crystal violet for 15 min, and photographed. Photographs were analyzed using ImageJ software (Ver 1.49, http://imagej.nih.gov/ij (accessed on 17 March 2019) to quantitate migration.

Invasion was evaluated similarly, except for a layer of Matrigel™ (1 mg/mL, 50 µL, BD Biosciences) which was used to coat the top of the insert membrane. Cells (4 × 10^4^) were seeded in the upper chamber and allowed to invade through the Matrigel™ layer for 24 h toward the laminin chemoattractant in the outer well. Inserts were then fixed, stained, and photographed as described for migration. Photographs were analyzed using ImageJ to quantitate invasion.

### 2.12. Anchorage-Independent Growth

A soft agar assay was utilized to assess for anchorage-independent growth. A base layer of 1% noble agar mixed with culture media was established in 60 mm culture dishes. AS WT or STRAP KO cells (1 × 10^4^) were plated in the top layer in the agar and culture media mixture. After 6 weeks, colonies were stained with crystal violet, imaged, and quantified using ImageJ.

### 2.13. RNA Sequencing and Analysis

Total cellular RNA was extracted from AS WT and AS STRAP KO cells using the RNAeasy kit (Qiagen) according to the manufacturer’s protocol. UAB Genomics Core performed sample quality control, library preparation, and RNA sequencing. The Agilent 2100 Bioanalyzer was used to assess the total RNA quality, which was followed by two rounds of poly A+ selection and conversion to cDNA. The NEBNext^®^ Ultra™ Directional RNA Library Prep Kit for Illumina^®^ library generation kit (New England Biolabs) was used per the manufacturer’s instructions. qPCR in a Roche LightCycler 480 with the Kapa Biosystems kit (Kapa Biosystems, Woburn, MA, USA) was used for library quantitation. The Illumina NextSeq500 was used to perform the sequencing using the latest versions of the sequencing reagents and flow cells with single-end 75 bp reads. Raw and processed data were deposited in the Gene Expression Omnibus (GEO, Accession GSE169322) [14].

STAR (version 2.7.3a) was used to align the raw RNA-Seq fastq reads to the human reference genome (GRCh38 p13 Release 32) from Gencode using parameters the following parameters: outReadsUnmapped Fastx; outSAMtype BAM SortedByCoordinate; outSAMattributes All --outFilterIntronMotifs RemoveNoncanonicalUnannotated [15]. Following alignment, Cufflinks (version 2.2.1) was used to assemble transcripts, estimate their abundances, and test for differential expression and regulation using parameters—library-type fr-firststrand-G–L [16]. Cuffmerge, which is part of Cufflinks, merged the Cufflinks transcripts across multiple samples using the default parameters. Cuffdiff found significant changes in transcript expression, splicing, and promoter usage using default parameters. For generating pathway analysis of biological processes, a data set containing gene identifiers and corresponding expression values was uploaded into Reactome Pathway Database [17]. Differentially expressed genes that met the fold change cutoff of ±2 were considered for the analysis. Each identifier is mapped to its corresponding molecule in the Reactome database and pathways identified at a false discovery rate (FDR) of 0.05. Entities ratio is defined as the proportion of Reactome pathway molecules represented in the dataset.

### 2.14. Real-Time PCR (qPCR)

iScript cDNA Synthesis kit (Bio-Rad) was used to synthesize cDNA with 1 μg of RNA used in a 20 μL reaction. The reverse transcription products were stored at −20 °C until further use. SsoAdvanced™ SYBR^®^ Green Supermix (Bio-Rad) was utilized according to the manufacturer’s protocol for quantitative real-time PCR (qPCR). Primers specific for Octamer-binding transcription factor 4 (Oct4), homeobox protein Nanog, and β-actin were utilized (Applied Biosystems, Foster City, CA, USA). Nestin primers (forward: 5′-TCCAGGAACGGAAAATCAAG-3′, reverse: 5′-GCCTCCTCATCCCCTACTTC-3′) were designed using Primer3 web version 4.1.0 [18] and examined for non-specific binding using BLAST (NCBI). qPCR was performed with 10 ng cDNA in 20 μL reaction volume. Amplification was performed using an Applied Biosystems 7900HT cycler (Applied Biosystems). Cycling conditions were 95 °C for 2 min, followed by 39-cycle amplification at 95 °C for 5 s and 60 °C for 30 s. β-actin was utilized as an internal control. Gene expression was calculated using the ΔΔCT method [19] and reported as mean fold change ± SEM.

### 2.15. CD133 Cell Surface Expression

AS WT or STRAP KO cells (1 × 10^6^) were labeled with CD133/1 (AC133)-APC (Miltenyi Biotec, San Diego, CA, USA) according to the manufacturer’s instructions. Unlabeled cells served as negative controls. The percent of cells positive for APC was determined via flow cytometry using the FACSCalibur™ Flow Cytometer (BD Biosciences) and analyzed using the FlowJo software (FlowJo, LLC FlowJo, Ashland, OR, USA).

### 2.16. Extreme Limiting Dilution Analysis

AS WT or STRAP KO cells were plated in conditioned media in 96-well plates with a decreasing number of cells in each row of 12 wells (1000 to 10 cells). After one week, each well was assessed for formation of tumorspheres. The number of wells containing spheres in each group was counted and analyzed using the extreme limiting dilution analysis (ELDA) software [20] and a plot of the log proportion of negative wells versus the number of cells plated was generated. The slope of the line is the estimated log-active sphere-forming fraction. Tables showing estimated and 95% confidence intervals for the 1/(stem cell frequency) for each group were also generated.

### 2.17. Animal Statement

Animal experiments were approved by the University of Alabama at Birmingham (UAB) Institutional Animal Care and Use Committee (IACUC-09363) and were conducted within institutional, national, and NIH guidelines and in compliance with the Animal Research: Reporting of In Vivo Experiments (ARRIVE) guidelines.

### 2.18. In Vivo Tumor Growth

AS WT or STRAP KO cells (1.8 × 10^6^ cells in 25% Matrigel™, Corning, Inc.) were injected into the right flank of 6-week-old female athymic nude mice (*n* = 9 per group, Envigo, Prattville, AL, USA). Tumors were measured three times weekly, and tumor volumes calculated with the formula [(width^2^ × length)/2] mm^3^ with width being the smallest measurement. After 21 days post-injection or when animals met IACUC parameters for euthanasia, the animals were humanely euthanized in their home cages with CO_2_ and cervical dislocation. Tumors were harvested and prepared for further study.

### 2.19. Immunohistochemistry

In order to evaluate proliferation in the tumors, immunohistochemistry staining for Ki67 [21] was performed. Formalin-fixed paraffin-embedded samples of AS WT and STRAP KO flank tumors were cut (5 μm sections), baked for 1 h at 70 °C, deparaffinized, rehydrated, and steamed. The sections were quenched with 3% hydrogen peroxide and blocked with PBS-blocking buffer (BSA, powdered milk, Triton X-100, PBS) for 30 min at 4 °C. Ki67 staining was completed by adding the primary antibody anti-Ki67 (rabbit polyclonal, 1:100, AB9260, Millipore Sigma) and incubated overnight at room temperature. After PBS washing, secondary antibody for rabbit (R.T.U. biotinylated universal antibody, Vector Laboratories, Burlingame, CA, USA) was added for 1 h at 22 °C. VECTASTAIN Elite ABC reagent (PK-7100, Vector Laboratories) and Metal Enhanced DAB Substrate (Thermo Fisher Scientific) was used to develop the staining reaction. Slides were counterstained with hematoxylin. For each run, a negative control (rabbit IgG, 1 μg/mL, EMD Millipore) was included. A board-certified pediatric pathologist (EMM), blinded to the treatment groups, evaluated the Ki67 staining which was quantified by counting the number of Ki67 positive cells per 500 cells in a representative section of each tumor [21]. The mean was calculated and results reported as mean ± SEM.

### 2.20. Statistical Analysis

ImageJ was utilized to perform densitometry of immunoblots with each protein band being normalized to the background (http://www.yorku.ca/yisheng/Internal/Protocols/ImageJ.pdf and the protein band in question then normalized to the internal control (vinculin or β-actin). Normalized bands were compared to that of WT cells. All experiments were performed with a minimum of three biologic replicates. Data were reported as mean ± SEM of separate experiments. Student’s *t* test or analysis of variance (ANOVA) were used where appropriate. Statistical significance was defined as *p* ≤ 0.05.

## 3. Results

### 3.1. STRAP Knockdown Decreased Proliferation, Growth, and Motility

**Figure 1:**
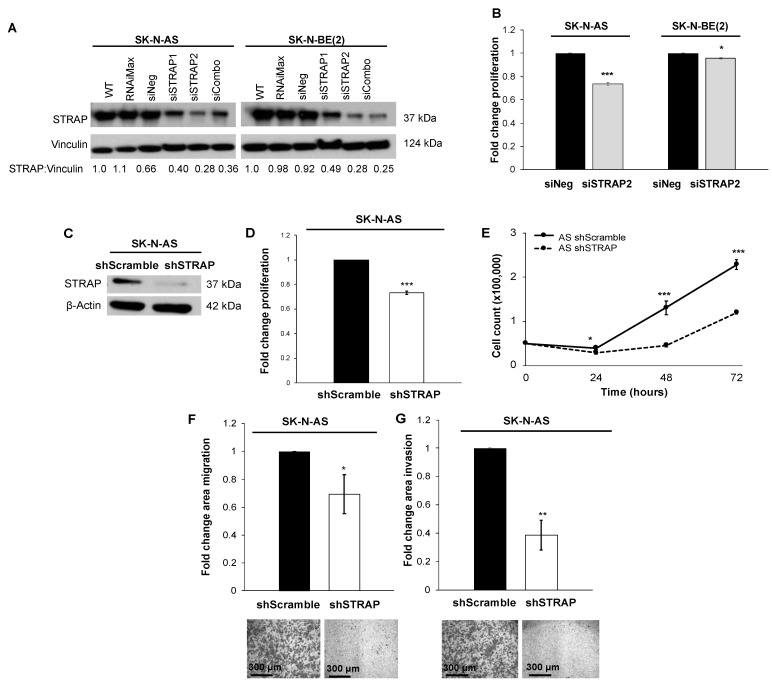
STRAP knockdown decreased neuroblastoma cell proliferation, growth, and motility. (**A**) SK-N-AS (AS) and SK-N-BE(2) (BE) cells were transfected with STRAP siRNA for 3 days. Immunoblotting of whole cell lysates revealed STRAP knockdown after transfection with siSTRAP1 or siSTRAP2 or both siRNAs (siCombo) compared to control siRNA (siNeg) or RNAiMax transfection reagent. Densitometry was used to quantify STRAP knockdown. Transfection with siSTRAP2 resulted in the most marked knockdown in STRAP expression in both the AS and BE cell lines. Therefore, siSTRAP2 was chosen for further studies. Vinculin was used as a loading control. (**B**) In both AS and BE cell lines, STRAP knockdown demonstrated a significant decrease in proliferation, measured by CellTiter 96^®^ assay, compared to control siNeg cells. (**C**) Stable transfection of shScramble (control) and shSTRAP was established in AS cells. Immunoblotting of whole cell lysates confirmed the knockdown of STRAP in the shSTRAP cells. Β-actin was used as a loading control. (**D**) Knockdown of STRAP with shRNA resulted in decreased proliferation in AS shSTRAP compared to AS shScramble cells. (**E**) The shScramble and shSTRAP cells were plated and counted at 24 h time points up to 72 h. The shSTRAP cells demonstrated significantly decreased growth over time compared to shScramble control cells. (**F**) To assess the effect of STRAP knockdown on motility, cells were seeded into modified Boyden chambers and allowed to migrate through an 8 µm micropore membrane for 24 h. There was a significant decrease in migration in shSTRAP cells compared to shScramble control cells. (**G**) Invasion was assessed similarly to migration with the addition of a layer of Matrigel™ to the top side of the insert. The shSTRAP cells demonstrated a significantly decreased ability to invade through the Matrigel™ layer compared to the shScramble control cells. Representative photomicrographs of stained migration and invasion inserts are shown in the graphs below and scale bars are shown in the bottom left corner of representative photographs. Experiments were repeated with at least three biologic replicates and data reported as mean fold change ± SEM. * *p* ≤ 0.05, ** *p* ≤ 0.01, and *** *p* ≤ 0.001.

Immunoblotting confirmed the presence of STRAP in five long-term passage neuroblastoma cell lines (Supplemental Figure S1). STRAP knockdown (KD) was accomplished with transient transfection of siRNA. Immunoblotting confirmed decreased STRAP protein expression in AS and BE cells following siSTRAP transfection (Figure 1A). Knockdown with siSTRAP2 was more marked than with siSTRAP1. STRAP expression was not affected by transfection with RNAiMax or siNeg (Figure 1A). STRAP band intensity was quantified with densitometry (Figure 1A). We wished to determine if STRAP KD affected neuroblastoma proliferation. We chose siSTRAP2 (SASI_Hs02-00343131) for these studies since it demonstrated the most knockdown in AS cells and in BE cells by densitometry (Figure 1A). STRAP KD via siRNA significantly decreased proliferation in the AS cells by 26 ± 1% (*p* ≤ 0.001) and in the BE cells by 4 ± 1% (*p* ≤ 0.05), when compared to the siNeg transfected cells (Figure 1B).

In order to further validate the siRNA findings, we next investigated STRAP knockdown (KD) using stable lentiviral transfection of AS cells with shScramble and shSTRAP plasmids. Due to the more rapid growth rate of the AS cell line versus BE, the focus of our phenotypic studies on STRAP were performed using AS cells to optimize experimentation and to produce the important conclusions discussed in this paper on the role of STRAP in neuroblastoma. Lentiviral transfection of AS cells resulted in successful STRAP KD (Figure 1C). The shSTRAP cells demonstrated a 27 ± 1% decrease in proliferation compared to the control shScramble cells (*p* ≤ 0.001, Figure 1D). When examining cell growth over 72 h, we found a significant decrease in growth over time in the shSTRAP cells compared to the shScramble controls (*p* ≤ 0.05, Figure 1E).

Other researchers have demonstrated the effects of STRAP on cancer cell motility [6,8,22]. In order to determine whether neuroblastoma cell motility was affected by STRAP KD, we utilized modified Boyden chamber assays to assess migration and invasion. The ability of the shSTRAP cells to migrate (65 ± 10% in shSTRAP cells compared to shScramble, *p* ≤ 0.05, Figure 1F) and invade (38 ± 10% in shSTRAP cells compared to shScramble, *p* ≤ 0.01, Figure 1G) was significantly decreased. These results indicate STRAP inhibition with shRNA affected motility in neuroblastoma.

### 3.2. STRAP KO Decreased Proliferation and Growth

**Figure 2:**
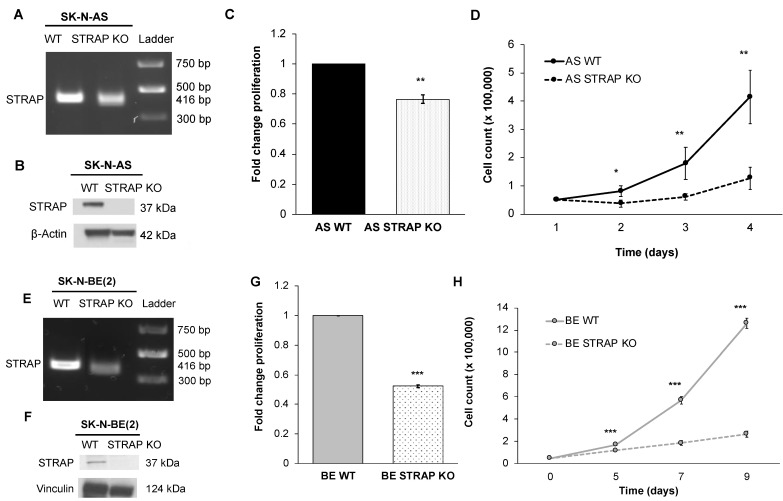
CRISPR-Cas9 gene knockout of STRAP decreased proliferation and growth in SK-N-AS and SK-N-BE (2) cells. (**A**) Gel electrophoresis was used to examine PCR products following amplification of genomic DNA isolated from clones transfected with STRAP gRNA and using STRAP primers. The STRAP band was found at the expected size of 416 bp in unedited SK-N-AS (AS) WT cells. The AS STRAP KO demonstrated two DNA fragment bands at a lower molecular weight than the WT, indicating a cut in the DNA following CRISPR-Cas9 gene editing. (**B**) Immunoblotting of whole cell lysates confirmed the absence of detectable STRAP protein in AS STRAP KO cells. (**C**) AS WT and STRAP KO cells were plated and proliferation was measured with CellTiter 96^®^ assay at 24 h. AS STRAP KO demonstrated significantly decreased proliferation compared to AS WT. (**D**) AS WT and AS STRAP KO cells were plated and counted for 72 h. The AS STRAP KO cells had significantly decreased cell growth over time compared to AS WT cells. (**E**) Similar to AS cells following CRISPR-Cas9 gene editing, a DNA gel confirmed the presence of the STRAP band at the expected 416 bp in SK-N-BE (2) (BE) WT cells and two DNA fragments in the BE STRAP KO cells. (**F**) Immunoblotting of whole cell lysates confirmed the absence of detectable STRAP protein in BE STRAP KO cells. Similar to AS STRAP KO cells, BE STRAP KO cells had decreased (**G**) proliferation and (**H**) growth over time compared to BE WT cells. Data reported as mean fold change ± SEM. Experiments were repeated with at least three biologic replicates. * *p* ≤ 0.05, ** *p* ≤ 0.01, and *** *p* ≤ 0.001.

Due to the potential for off-target effects seen with si/shRNA technology [23], we wished to determine the effect of decreased STRAP on the neuroblastoma cell phenotype following knockout of the STRAP gene. Using CRISPR-Cas9 gene editing technology, we established stable STRAP KO cells in two neuroblastoma cell lines, AS and BE. DNA gel showed the unedited WT DNA band to be at the expected 416 bp while the STRAP KO DNA band yielded lower molecular weight DNA fragments, confirming a cut in the DNA in both cell lines (Figure 2A,E). DNA fragments were excised and sent for Sanger sequencing. Using BLAST (https://pubmed.ncbi.nlm.nih.gov/22708584/, sequencing revealed that the unedited WT DNA aligned to the human reference gene, while the STRAP KO band possessed a gap which corresponded to the position of the STRAP gRNA that had been introduced. These findings confirmed the successful genetic knockout of STRAP in AS (Supplemental Figure S2A) and BE cells (Supplemental Figure S3A). In addition, immunoblotting confirmed the resulting absence of STRAP protein expression in the STRAP KO cells of both cell lines (Figure 2B,F).

Other investigators have shown the effects of STRAP on cancer cell growth [11,22]. Proliferation, as measured by CellTiter 96^®^, demonstrated a 23% decrease in the STRAP KO compared to AS WT cells (*p* ≤ 0.01, Figure 2C). Examining cell growth over time, STRAP KO had significantly decreased growth compared to AS WT cells (*p* ≤ 0.05, Figure 2D). In order to further validate the role of STRAP in these functions, we performed the same experiments in an additional neuroblastoma cell line, BE, with stable STRAP CRISPR-Cas9 KO. Similar to the findings in the AS cells, STRAP KO in BE cells resulted in decreased proliferation (52%, *p* ≤ 0.01, Figure 2G) and growth (*p* ≤ 0.01, Figure 2H) compared to BE WT cells.

### 3.3. STRAP KO Resulted in Failure to Progress through the Cell Cycle

**Figure 3:**
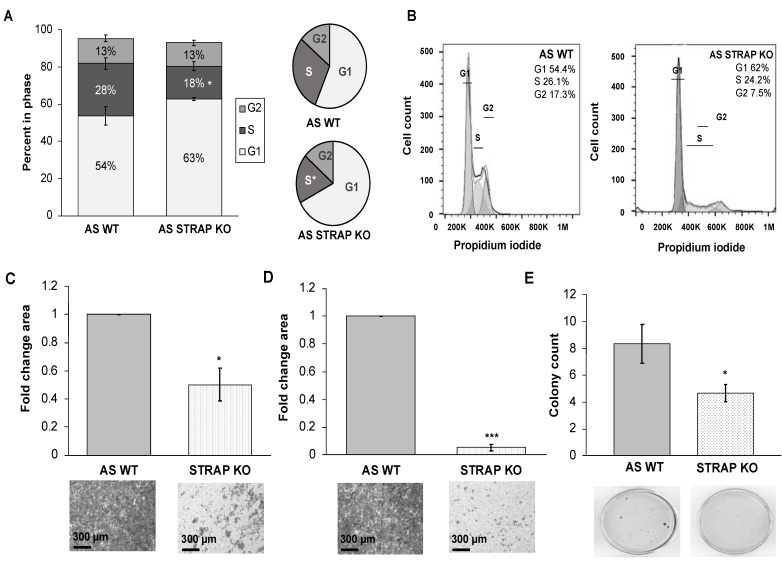
STRAP knockout diminished cell cycle progression and motility. (**A**) AS WT and AS STRAP KO cells were serum starved for 24 h then plated in routine culture media and stained with propidium iodide after 24 h. Flow cytometry was used to analyze progression through the cell cycle. AS STRAP KO had significantly decreased the percentage of cells in the S phase and an associated increased percentage of cells was observed in the G1 phase. Values represent mean percentage of cells in phase from three independent biologic experiments. (**B**) Representative histograms of AS WT and AS STRAP KO cells from a single experiment. (**C**) AS WT and AS STRAP KO cells were allowed to migrate for 24 h through a micropore membrane. Representative photomicrographs of migration inserts stained with crystal violet are shown beneath the graphs. Scale bars are shown in the bottom left corner of the representative photographs. AS STRAP KO cells had a significant decrease in migration compared to AS WT cells. Migration was reported as mean fold change area of migration ± SEM. (**D**) Similarly, AS STRAP KO cells demonstrated significantly decreased invasion after 24 h compared to AS WT. Representative photomicrographs of stained invasion inserts are shown below the graphs and scale bars are shown in the bottom left corner of representative photographs. Invasion was reported as mean fold change area of invasion ± SEM. (**E**) In order to assess for anchorage-independent growth, AS WT and AS STRAP KO cells were grown in a soft agar for 6 weeks and colonies were stained and quantified. Colony count was significantly lower in AS STRAP KO than in AS WT cells, which indicates a decrease in anchorage-independent growth in the AS STRAP KO cells. Data reported as mean colony count ± SEM. Experiments were repeated with at least three biologic replicates. * *p* ≤ 0.05 and *** *p* ≤ 0.001.

In order to investigate a potential mechanism by which STRAP KO decreased cell proliferation, we examined the cell cycle. There was a significant decrease in the percentage of AS STRAP KO cells in the S phase (17.5% vs. 28.2%, STRAP KO vs. AS WT, *p* ≤ 0.01, Figure 3A) and an increase in the percentage of AS STRAP KO cells in the G1 phase (62.7% vs. 53.8%, STRAP KO vs. AS WT, *p* = 0.08, Figure 3A). Data from three biologic replicates are presented in tabular form in Supplemental Table S1. The decrease in S phase provides evidence that STRAP KO cells do not progress through the cell cycle as well as their WT counterparts. These results support our findings of a decrease in proliferation in the STRAP KO cells (Figure 2C). Representative histograms of AS WT and AS STRAP KO cells from a single experiment are presented in Figure 3B.

### 3.4. STRAP KO Decreased Migration and Invasion

STRAP inhibition with shRNA technology decreased neuroblastoma motility (Figure 1E,F). We wished to determine whether the absence of STRAP would similarly affect the motility of neuroblastoma cells. As in the shRNA experiments, we employed modified Boyden chamber assays for migration and invasion. AS STRAP KO cells had significantly decreased migration (50% of cells migrated compared to AS WT, *p* ≤ 0.05, Figure 3C) and invasion (5% of cells invaded compared to AS WT, *p* ≤ 0.001, Figure 3D). Representative photographs of the migration and invasion inserts are displayed below the graphs (Figure 3C,D).

Anchorage-independent growth is another common metric to determine the metastatic potential of cancer cells [24]. In order to assess anchorage independent growth, AS WT and AS STRAP KO cells were cultured in anchorage-independent conditions using soft agar assays. AS STRAP KO cells formed fewer colonies than AS WT under these conditions (*p* ≤ 0.05, Figure 3E). These results suggest STRAP affected neuroblastoma cell motility and potential for metastasis.

### 3.5. Transcriptomic Effects of the Loss of STRAP

**Figure 4:**
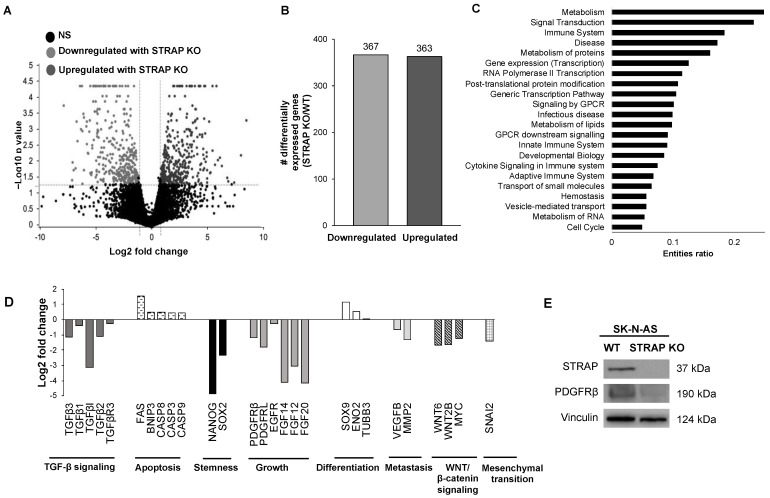
Knockout of STRAP resulted in changes in expression of oncogenic genes. (**A**) RNA sequencing was performed on AS WT and AS STRAP KO cells. A volcano plot of expressed genes is shown with the dotted horizontal line representing a *p* value of 0.05 and the vertical dotted lines representing a Log2 fold change of ±1. Dark grey dots represent genes that were significantly upregulated by at least two-fold following STRAP KO and the light grey dots representing those that were significantly downregulated by at least two-fold. Non-significant (NS) genes (*p* value > 0.05) are shown in black. (**B**) Differentially expressed genes following STRAP KO of AS neuroblastoma cells are presented in a bar graph. There were 367 genes that were significantly downregulated while 363 genes were significantly upregulated in AS STRAP KO cells. (**C**) Reactome Pathway Database was used to evaluate downregulated genes in AS STRAP KO and pathways with the highest entities ratio presented. The ratio indicates the number of gene entities from the dataset that map to the Reactome pathway divided by the total number of entities in that pathway. Genes downregulated following STRAP KO were associated with metabolic pathways, cell signaling transduction, gene transcription, and cell cycle progression. (**D**) Fold change expression of selected genes in STRAP KO compared to AS WT cells is shown. STRAP KO of AS neuroblastoma cells downregulated genes involved in stemness, growth, metastasis, mesenchymal transition, and multiple oncogenic signaling pathways such as TGF-β and WNT/β-catenin signaling. On the contrary, genes linked to apoptosis and differentiation were upregulated following STRAP KO. (**E**) PDGFRβ protein expression was evaluated with immunoblotting and found to be decreased in AS STRAP KO cells compared to AS WT cells.

In order to evaluate the effect of STRAP KO on neuroblastoma at the transcriptomic level, we utilized RNA-seq to investigate changes in gene expression in AS STRAP KO compared to AS WT cells. We utilized a volcano plot to represent the respective differences in gene expression in AS STRAP KO compared to AS WT cells (Figure 4A). Based on RNA-seq, there was a significant downregulation by at least two-fold of 367 genes in AS STRAP KO vs. AS WT, while 363 genes were significantly upregulated by at least two-fold (*p* ≤ 0.05, Figure 4B). We utilized the Reactome Pathway Database to evaluate pathways associated with the genes downregulated by at least two-fold in the AS STRAP KO cells and found that genes downregulated following STRAP KO were associated with metabolic pathways, cell signaling transduction, gene transcription, and cell cycle progression (Figure 4C). We further investigated genes commonly associated with the malignant phenotype, such as apoptosis [25] and metastasis [26,27]. We found that STRAP KO downregulated genes were involved in stemness, metastasis, and multiple oncogenic signaling pathways such as TGF-β and WNT/β-catenin signaling, while genes associated with apoptosis and differentiation were upregulated in the AS STRAP KO cells (Figure 4D). We were also interested in genes associated with promoting tumor growth [28] and found several that were downregulated in the AS STRAP KO cells, notably platelet-derived growth factor receptor ß (PDGFRβ) (Figure 4D). PDGFRβ is a molecule of interest in neuroblastoma [29,30,31]. We examined PDGFRβ protein expression using immunoblotting and found a decrease in PDGFRβ expression in the AS STRAP KO compared to AS WT cells (Figure 4E), further validating our findings at the protein level.

### 3.6. STRAP KO Decreased Tumor Cell Stemness

**Figure 5:**
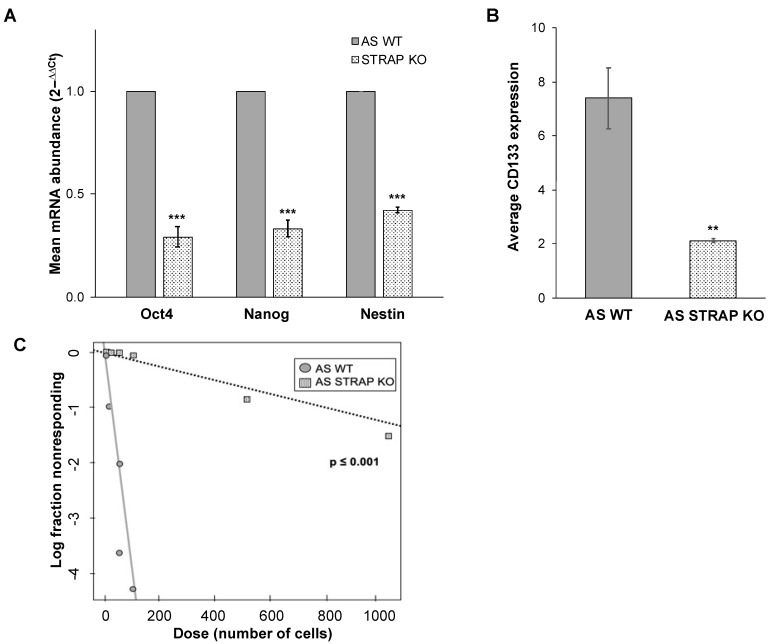
STRAP knockout decreased neuroblastoma cell stemness. (**A**). Quantitative real-time PCR was utilized to evaluate mRNA abundance of the common neuroblastoma stem cell markers Oct4, Nanog, and Nestin in AS WT and AS STRAP KO cells. AS STRAP KO cells had significantly decreased mRNA abundance of these stemness markers compared to AS WT. Gene expression was normalized to β-actin and calculated as fold change to AS WT using the ∆∆Ct method. (**B**) AS WT and AS STRAP KO cells were stained with CD133 antibody and CD133 cell surface expression was determined using flow cytometry. STRAP KO cells had significantly decreased CD133 expression compared to WT cells. (**C**) In order to further evaluate stemness, an extreme limiting dilution analysis was utilized to assess tumorsphere formation. Cells were plated in conditioned media in non-adherent conditions at decreasing cell concentrations per well. Wells with tumorspheres present were counted and analyzed. AS STRAP KO cells had significantly decreased ability to form tumorspheres compared to AS WT cells. Data reported as mean ± SEM. Experiments were repeated with at least three biologic replicates. ** *p* ≤ 0.01, *** *p* ≤ 0.001.

STRAP increased cancer cell stemness in colorectal [11] and hepatocellular carcinoma [32]. After examining the transcriptomic data described, we found gene expression of the stemness markers NANOG and SOX 2 to be downregulated in the STRAP KO cells (Figure 4D). Therefore, we wished to determine if STRAP played a role in maintaining a stem cell-like phenotype in neuroblastoma. We performed qPCR to examine the mRNA abundance of the common neuroblastoma stem cell markers, Oct4, Nanog [33], and Nestin [34]. The AS STRAP KO cells had a decreased abundance of mRNA of these three stem cell markers compared to AS WT cells (*p* ≤ 0.001, Figure 5A). We also investigated another feature associated with neuroblastoma stemness, which is the expression of the cell surface marker CD133 [35], using flow cytometry. CD133 cell surface expression decreased by 71% in AS STRAP KO compared to AS WT cells (*p* ≤ 0.01, Figure 5B).

The ability of cells to form tumorspheres in low attachment serum-free conditions is a hallmark of stemness. An extreme limiting dilution analysis was employed to examine the effect of AS STRAP KO on tumorsphere formation. AS STRAP KO cells possessed significantly decreased ability to form tumorspheres compared to AS WT cells (Figure 5C). The stem cell frequency of AS STRAP KO cells was 1/796 (95% confidence interval of 1/1054–1/601) vs. 1/24 (1/30–1/20) in AS WT (*p* ≤ 0.001, Supplemental Table S2). These results demonstrate STRAP KO decreases stemness in neuroblastoma.

### 3.7. Re-Introduction of STRAP Demonstrated Rescue of the Malignant Phenotype

In order to alleviate concerns of off-target effects resulting in the phenotype noted with the CRISPR-Cas9 generated STRAP KO cells, we performed STRAP rescue experiments. AS STRAP KO cells (3 × 10^3^ cells) were plated and transfected with either FuGENE^®^, FuGENE^®^ and EV pc-DNA plasmid, or FuGENE^®^ and STRAP pc-DNA plasmid. Immunoblotting confirmed the absence and subsequent rescue of STRAP protein expression in the AS STRAP KO cells (Supplemental Figure S4A). In order to assess the phenotype of STRAP rescue cells, we evaluated proliferation. AS STRAP KO cells had significantly decreased proliferation (*p* ≤ 0.05, Supplemental Figure S4B), which is consistent with data shown in the current study (Figure 3D). The re-introduction of STRAP (rescue) in the AS STRAP KO cells increased proliferation relative to the baseline level seen in AS WT cells, which demonstrates reconstitution of the malignant phenotype by re-introducing STRAP in the STRAP KO cells (Supplemental Figure S4B). These findings demonstrate STRAP conferred the phenotype seen in the AS STRAP KO cells.

### 3.8. STRAP KO Decreased Tumor Growth In Vivo

**Figure 6:**
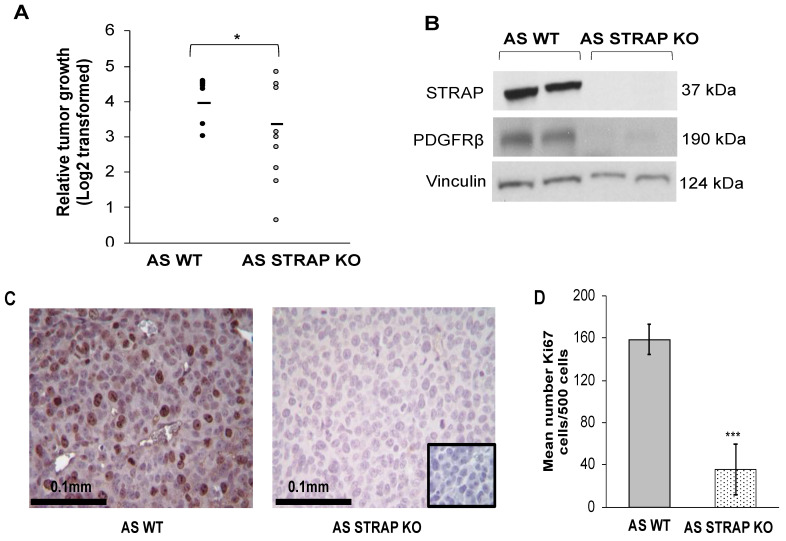
STRAP knockout decreased neuroblastoma tumor growth and proliferation in vivo. (**A**) AS WT or AS STRAP KO cells (1.8 × 10^6^) were injected into the flanks of athymic nude mice (*n* = 9 per group). Tumor volumes were measured three times a week and tumors were harvested when IACUC parameters were met. STRAP KO tumors had a significantly decreased relative tumor growth compared to WT tumors. Results are reported as mean (Log2 transformed) ± SEM. (**B**) Using immunoblotting of tumor lysates, STRAP KO was confirmed in the STRAP KO tumors. Immunoblotting was also utilized to investigate PDGFRβ protein expression in the tumors and demonstrated decreased expression in the AS STRAP KO tumors compared to AS WT tumors. (**C**) Immunohistochemistry staining for Ki67, which is a marker for proliferation, was used on formalin-fixed paraffin-embedded AS WT and AS STRAP KO tumors. Representative pictures of Ki67 stained slides from each group are shown. A negative IgG control was included in each run (inset, right lower corner). (**D**) The number of Ki67 positive cells were counted per 500 cells. AS STRAP KO tumors had significantly decreased Ki67 positive cells compared to AS WT, indicating decreased proliferation. Results are reported as mean ± SEM. * *p* ≤ 0.05 and *** *p* ≤ 0.001.

Based on the in vitro data, we proceeded to an in vivo model. AS WT or AS STRAP KO cells (1.8 × 10^6^ cells in Matrigel™) were injected into the flank of athymic nude mice (*n* = 9 per group). Tumors were measured three times per week with calipers and tumor volumes were calculated as described. Animals with AS STRAP KO tumors had significantly decreased relative tumor growth (Figure 6A) and mean tumor volume (Supplemental Figure S5) compared to those with AS WT tumors. Immunoblotting confirmed the knockout of STRAP in the tumors from the animals injected with AS STRAP KO cells (Figure 6B). Similar to the in vitro studies AS STRAP KO tumors (Figure 6B) exhibited decreased protein expression of PDGFRβ, which is known to promote tumor growth in neuroblastoma [31]. Utilizing immunohistochemistry to detect Ki67, which is a marker of proliferation, we found that AS STRAP KO tumors had significantly less Ki67 staining than AS WT tumors and this signifies decreased proliferation (36 ± 24 vs. 159 ± 14 Ki67 positive cells out of 500 total cells, AS STRAP KO vs. AS WT, *p* ≤ 0.001, Figure 6C,D). IgG negative staining control was completed with each IHC run and is shown in the following (Figure 6C, right lower corner inset).

## 4. Discussion

High-risk neuroblastoma continues to carry a poor prognosis and continued investigations are crucial to improve our understanding of the disease. STRAP is overexpressed in several malignancies including another pediatric cancer, osteosarcoma [7,8,32,36]. Recently, Jin et al. found that STRAP functions in neuronal development and STRAP knockout in Xenopus resulted in neural tube defects [37]. These findings were relevant to the present study, since neuroblastoma is an embryologic tumor derived from neural crest cells [2]. These previous studies supported the investigation of the potential for oncogenic function of STRAP in neuroblastoma. In the current study, we demonstrated that STRAP knockdown decreased neuroblastoma proliferation, growth, and motility. By establishing two stable neuroblastoma cell lines with the genetic knockout of STRAP and examining their malignant phenotype as well as the effect on the transcriptome, we provide evidence to suggest a role for STRAP in promoting neuroblastoma tumorigenicity in vitro and in vivo as well as maintaining the stem cell-like phenotype.

RNA interference is a method for transiently inhibiting gene expression and/or translation by targeting the corresponding RNA. Small interfering RNA (siRNA) and short hairpin RNA (shRNA) are two applications of RNA interference which have been utilized to achieve specific knockdown of a specific protein [38]. STRAP siRNA knockdown decreased migration and invasion of osteosarcoma cells [8]. However, Wu and colleagues found that knockdown of STRAP utilizing siRNA promoted hepatocellular carcinoma tumorgenicity in vitro and in vivo [36], suggesting that STRAP’s role in cancer may not be tumor-specific. We initially investigated the knockdown of STRAP by utilizing siRNA in two neuroblastoma cell lines, which are AS (MYCN non-amplified) and BE (MYCN amplified), and found that STRAP knockdown resulted in decreased proliferation. These findings led us to further explore the role of STRAP in neuroblastoma.

Although siRNA and shRNA may achieve similar knockdown, the mechanism of action is different. SiRNA is a transient transfection, whereas shRNA carrying cells may be selected with an antibiotic for stable transfection. It is hypothesized that shRNA may have higher potency and fewer off-target effects than siRNA [38], enabling us to investigate the knockdown of STRAP utilizing shRNA in addition to siRNA. Datta et al. demonstrated decreased tumorigenicity in colorectal cancer cells following transfection with STRAP shRNA [6]. Similarly, we found that shRNA-mediated stable knockdown of STRAP resulted in decreased neuroblastoma cell proliferation, growth, and motility. The same investigators also demonstrated an increase in the sensitivity to 5-FU and oxaliplatin in colorectal cancer following shRNA STRAP knockdown [11]. Studying the effects of STRAP knockdown in combination with neuroblastoma chemotherapeutics will be an exciting avenue of future studies.

RNA interference has several limitations including a decrease in expression of the protein of interest, but not a total absence of that protein [39]. Due to these limitations, we proceeded to investigate the genetic knockout of STRAP. Using CRISPR-Cas9 gene editing technology, we established a stable cell line of STRAP KO cells in the neuroblastoma cell line SK-N-AS. Wang et al. also utilized CRISPR-Cas9 gene editing technology to establish a stable line of STRAP KO cells in hepatocellular carcinoma and demonstrated that STRAP KO led to decreased Wnt/β-catenin signaling, which was associated with decreased colony formation and stemness markers in vitro [32]. In order to further validate our findings of STRAP’s role in neuroblastoma, we established an additional CRISPR-Cas9 stable knockout of STRAP in SK-N-BE (2) human neuroblastoma cells. We found that STRAP KO resulted in decreased growth, proliferation, motility, and anchorage-independent growth.

Using the findings from genetic sequencing of AS STRAP KO and AS WT cells, we found that numerous pathways were affected by the loss of STRAP. Specifically, pathways with the highest entities ratio that were associated with downregulated genes following the STRAP KO included those involved in metabolism, signal transduction, and cell cycles. We also found genes associated with stemness, growth, metastasis, and the known oncogenic pathways previously shown to be associated with STRAP by other investigators, such as TGF-β [22] and WNT/β-catenin [6], to be downregulated while apoptotic and differentiation genes were upregulated in the STRAP KO cells. Many of these pathways are important in neuroblastoma. Prior investigators showed that by stimulating neuroblastoma with TGF-β, there was an increase in cell migration and invasion [40]. Similarly, Tran et al. demonstrated the restoration of natural killer cells’ cytotoxicity following treatment with a TGF-β inhibitor in neuroblastoma [41]. SiRNA knockdown of the WNT/β-catenin signaling pathway has been shown to decrease neuroblastoma viability, growth [42], and motility [43]. Therefore, the inhibition of these oncogenic signaling pathways observed with STRAP KO provides support for the inhibition of STRAP in neuroblastoma as a therapeutic strategy.

Platelet-derived growth factor receptors (PDGFRs) have been associated with growth, angiogenesis, cell viability, and proliferation in numerous malignancies, including glioma, prostate, breast, and pancreatic cancers [44]. By inhibiting PDGFRβ signaling, other investigators decreased cell viability, increased apoptosis, and induced cell cycle arrest in mesothelioma [45]. In colorectal cancer, lower PDGFRβ expression was associated with better prognosis and PDGFRβ knockdown using siRNA resulted in decreased colorectal cancer proliferation, growth, and invasion [46]. PDGFRβ was one of the downregulated genes observed with AS STRAP KO and we confirmed decreased protein expression in the AS STRAP KO cells. These studies provide evidence that STRAP may exert its phenotypic effects on cell proliferation and growth in neuroblastoma through its effect on expression of PDGFRβ. PDGFRβ has become a receptor of interest in neuroblastoma. Targeting PDGFRs with imantinib, which is a tyrosine kinase inhibitor, decreased neuroblastoma survival and proliferation as well as enhanced apoptosis when combined with doxorubicin [47]. Similarly, other investigators inhibited neuroblastoma growth in vivo and decreased tumor angiogenesis using SU11657, a tyrosine kinase inhibitor that targets PDGFRs [30]. In the current study, when STRAP was knocked out, we observed a decrease in PDGFRβ which suggests the presence of a potential mechanism by which STRAP may promote the malignant phenotype.

Stem cell-like cancer cells (SCLCCs) are a subpopulation of cancer cells that have been shown to be important for neuroblastoma progression, therapeutic resistance, and disease recurrence [34]. Therefore, therapies that target SCLCCs could greatly affect the course of the disease. STRAP has been implicated in maintaining colorectal cancer cell stemness. Jin and colleagues found that STRAP inhibition with shRNA decreased CD133 positive colorectal cancer cells and tumorsphere forming ability [11]. STRAP promoted the stem cell-like characteristics of colorectal cancer cells by activating the NOTCH pathway and the silencing of STRAP in these cells resulted in decreased stem cell phenotype [11]. In addition, STRAP knockout in hepatocellular carcinoma decreased mRNA abundance of stemness markers and liver progenitor genes, including AXIN2, LGR5, CD133, and CD44 [32]. In the current study, genetic evaluation demonstrated a downregulation of genes associated with neuroblastoma stemness following the knockout of STRAP. Further phenotypic evaluation of AS STRAP KO cells demonstrated a significant decrease in mRNA abundance of stemness markers, a decrease in CD133 cell surface expression, and decreased ability to form tumorspheres; all of these indicate that STRAP plays a role in promoting neuroblastoma cancer cell stemness.

Other investigators have examined STRAP knockdown and its effects on in vivo tumor growth. In colorectal cancer, STRAP knockdown with shRNA led to decreased tumor growth [11] and metastasis [6] in vivo. In addition, the knockdown of STRAP with shRNA in hepatocellular carcinoma resulted in decreased tumorigenicity and metastasis [36]. To our knowledge, the current study is the first to investigate the effect of STRAP knockdown and CRISPR KO on neuroblastoma and the first investigation of STRAP CRIPSPR KO on neuroblastoma tumor growth in vivo. Similar to results described in other in vivo studies, we demonstrated that the genetic knock out of STRAP resulted in decreased neuroblastoma tumor growth with significantly decreased proliferation in the KO tumors.

One limitation of CRISPR-Cas9 genetic knockout is the possibility for off-target activity or non-specific gene editing [12,48,49]. Validation of the target gene’s role in the observed phenotype may be achieved by reintroducing the knocked-out gene in a rescue experiment. Other investigators have utilized this method. Chen and colleagues showed that CRISPR-Cas9 knockout of EZH2 in neuroblastoma resulted in decreased cell viability. They subsequently re-introduced EZH2, resulting in the rescue of the phenotype observed following EZH2 deletion. These findings confirmed EZH2′s importance in neuroblastoma survival [50]. In the present study, STRAP re-expression in the AS STRAP KO cells restored the phenotype to that observed in AS WT cells.

In the current study, we demonstrate that the knockdown and knockout of STRAP resulted in a decrease in the malignant phenotype in neuroblastoma. Our pre-clinical findings support the potential use of STRAP as a therapeutic target; however, there are currently no STRAP inhibitors available. Therefore, collaborations and development of inhibitors targeting STRAP are areas of interest and future studies. Interestingly, knocking down STRAP using siRNA had a more drastic effect on proliferation in AS cells compared to BE. One explanation for these findings is the difference in MYCN amplification in the two cell lines, which is related with a more aggressive phenotype in neuroblastoma [51]. An association between STRAP and MYCN has not yet been explored, providing us an additional avenue for future studies. STRAP is overexpressed in other cancers and has been associated with a more aggressive phenotype [6,8]; this provides an exciting avenue for future work to investigate STRAP expression levels in patient neuroblastoma specimens as a potential predictor for high-risk disease.

## 5. Conclusions

The data presented in the current study provide evidence that the inhibition of STRAP led to decreased neuroblastoma viability, proliferation, and motility in vitro and decreased tumor growth in vivo. Furthermore, we demonstrated STRAP’s role in promoting cancer cell stemness as STRAP KO resulted in decreased mRNA abundance of stemness markers, CD133 expression, and tumorsphere formation. These findings provide an exciting avenue to continue the investigation of STRAP’s role in neuroblastoma oncogenesis and as a potential therapeutic target for this disease.

## Data Availability

Raw and processed data were deposited in the Gene Expression Omnibus (GEO, Accession GSE169322).

## Supplementary Materials

The following are available online at https://www.mdpi.com/article/10.3390/cancers13133201/s1, Figure S1: STRAP protein is present in neuroblastoma cell lines. Figure S2: Sanger sequencing confirmed successful knockout of STRAP in SK-N-AS cells. Figure S3: Sanger sequencing confirmed successful knockout of STRAP in SK-N-BE(2) cells. Figure S4: Re-introduction of STRAP DNA rescued the malignant phenotype in STRAP knockout cells. Figure S5: STRAP knockout decreased neuroblastoma tumor growth in vivo. Table S1: STRAP knockout diminished progression through the cell cycle. Table S2: STRAP knockout decreased neuroblastoma tumorsphere formation.
