# Supplementary material for: Serine-Threonine Kinase Receptor-Associated Protein (STRAP) Knockout Decreases the Malignant Phenotype in Neuroblastoma Cell Lines
Source: Cancers (Basel). 2021 Jun 26;13(13):3201. doi: 10.3390/cancers13133201 (PMC8268080; doi:10.3390/cancers13133201)
Supplement: Supplementary file 1 [file cancers-13-03201-s001.zip › cancers-1279935 Supplementary Materials(6.29).pdf]

Supplementary Materials:

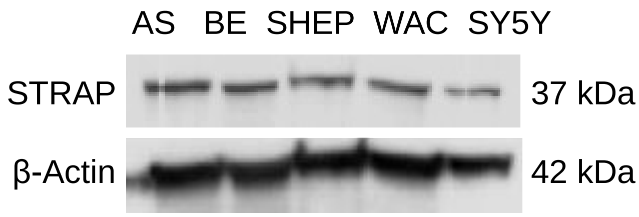

Supplemental Figure 1S: STRAP protein is present in neuroblastoma cell lines. A. Immunoblotting of whole cell lysates from SK-N-AS (AS), SK-N-BE(2) (BE), SHEP, WAC2 (WAC), and SH-SY5Y (SY5Y) cells revealed STRAP protein expression in all 5 long-term passage neuroblastoma cell lines. β-actin was used as a loading control.

A

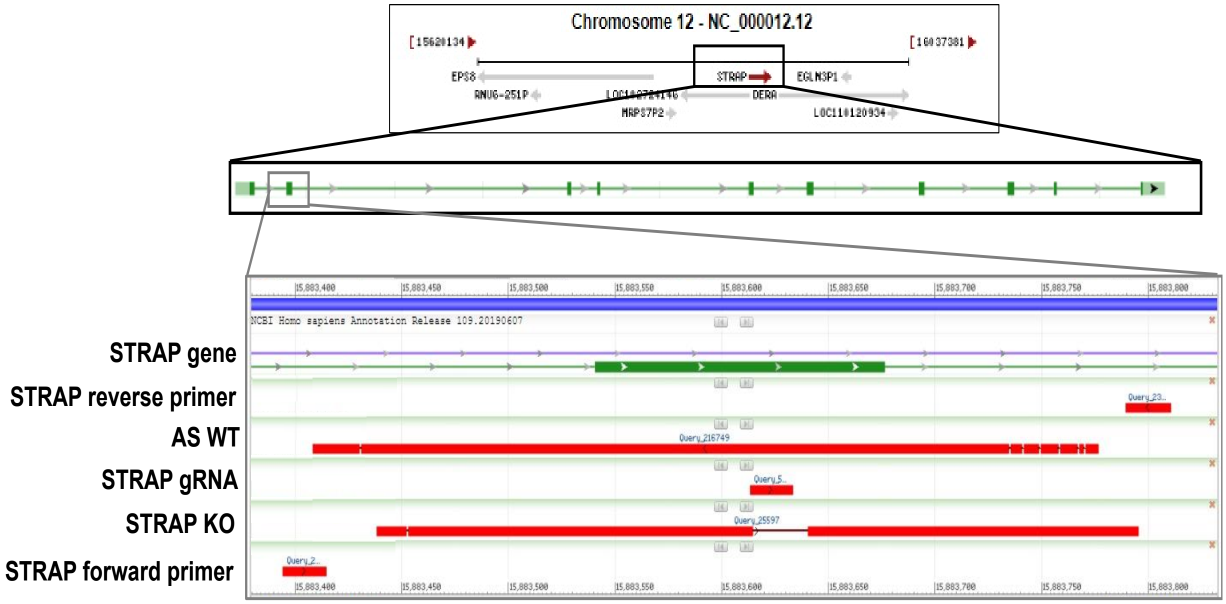

B

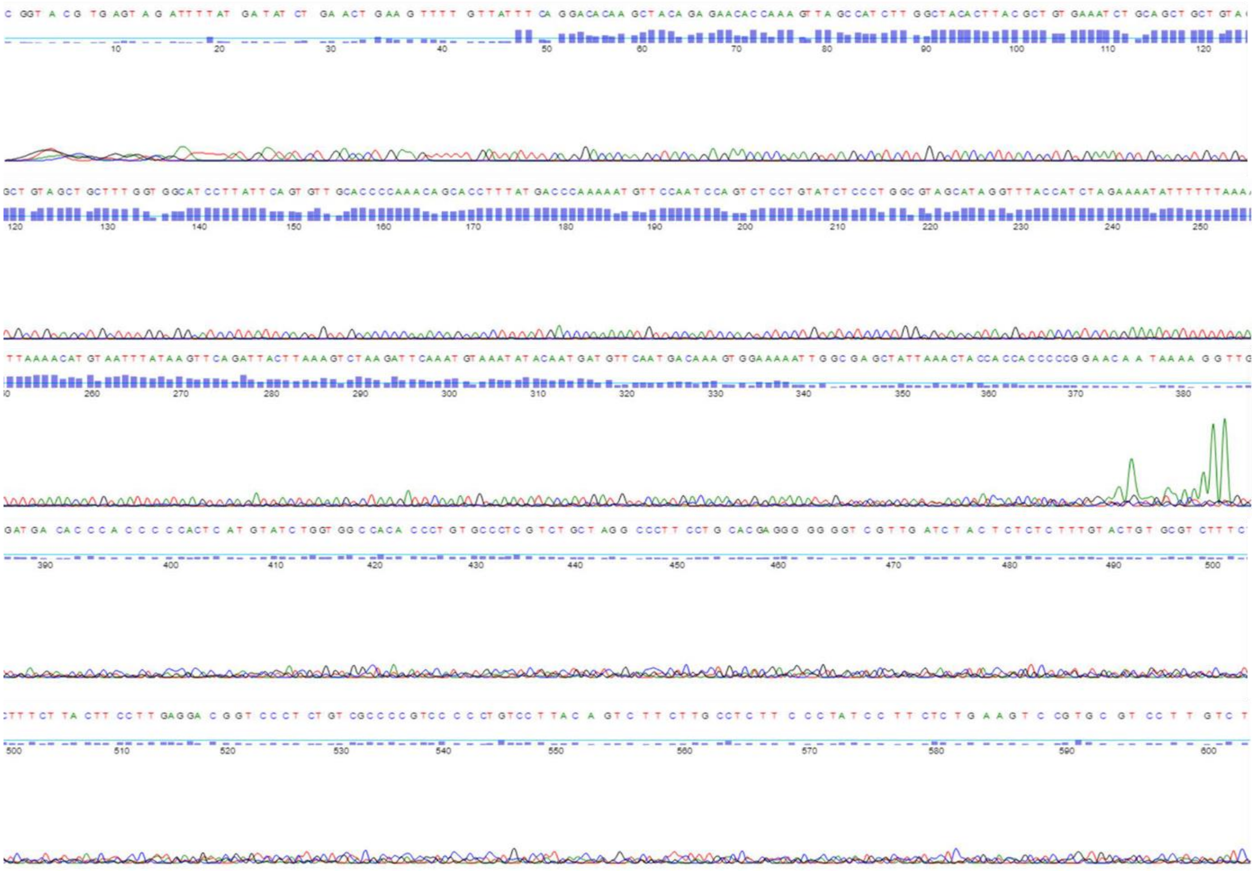

C

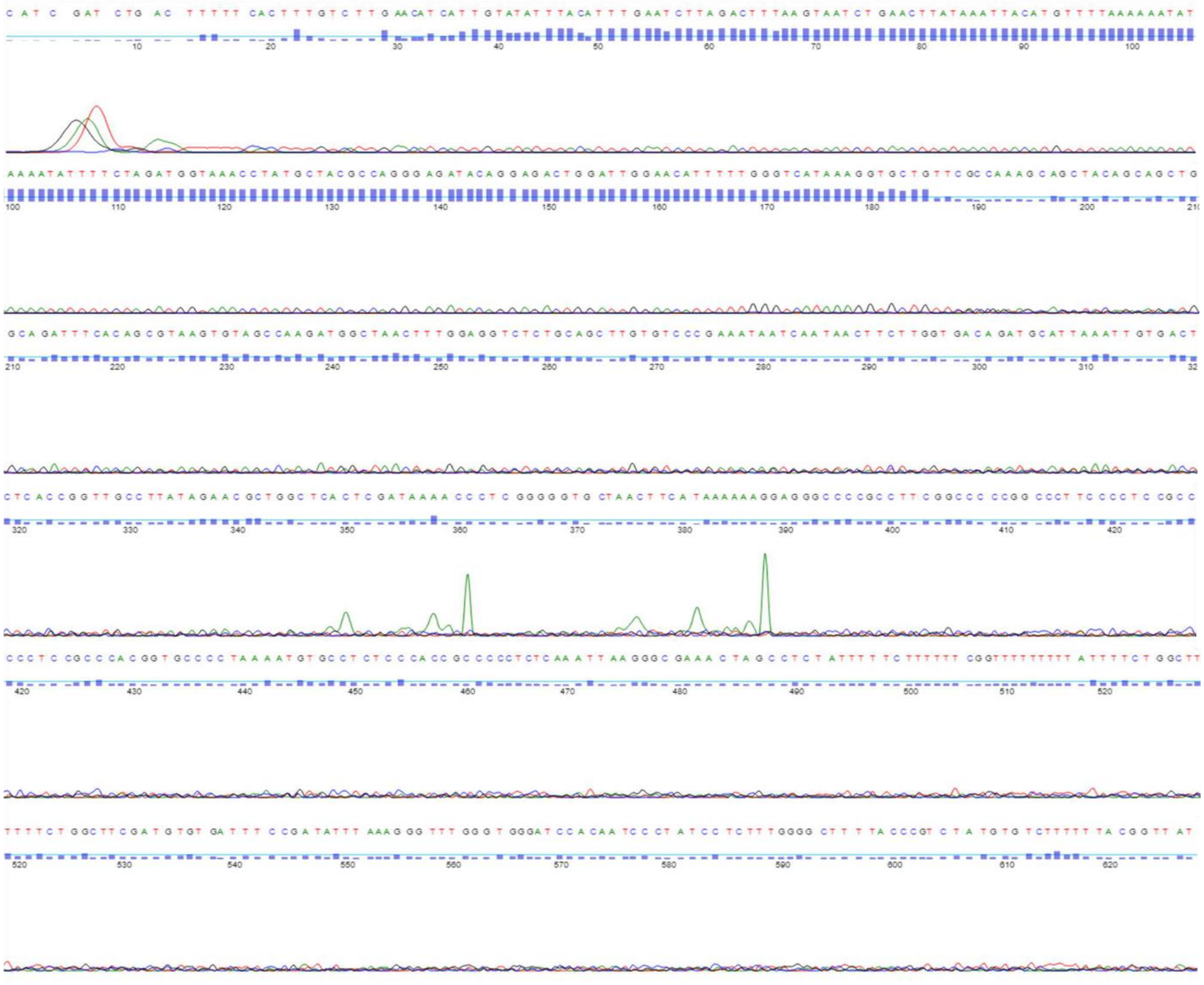

Supplemental Figure S2: Sanger sequencing confirmed successful knockout of STRAP in SK-N-AS cells. (A) Location and structure of the STRAP gene on chromosome 12 is depicted at the top (from 5′ to 3′) with exons indicated by dark green boxes. The region of interest (5′ to 3′ of exon 2) is shown in the zoomed in (boxed) region. Following amplification with STRAP primers, PCR products were assessed using gel electrophoresis. DNA fragments were purified from the DNA gel and nucleotide sequence analyzed by Sanger sequencing. Using BLAST, the sequencing results of both the SK-N-AS wild-type (WT) and STRAP knockout (KO) DNA were aligned to the human reference sequence. STRAP KO demonstrated a deletion (~20 bp) that corresponded to the location of the STRAP guide RNA (gRNA). The designed gRNA and the STRAP primer pair (forward and reverse primers) were also aligned, and their position is shown in relation to the sequencing results. (B, C) Chromatograms of AS WT (B) and AS STRAP KO (C) resulting from Sanger sequencing.

A

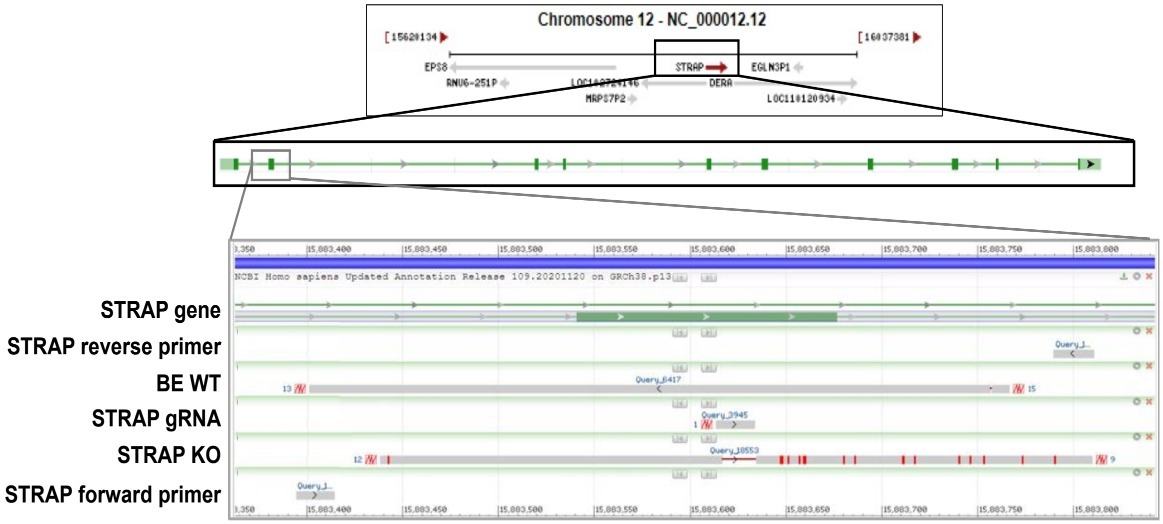

Chromosome 12 - NC_000012.12
EPS8
STRAP
EGLN3P1
DERA
MRPS7P2
STRAP gene
STRAP reverse primer
BE WT
STRAP gRNA
STRAP KO
STRAP forward primer
NCBI Homo sapiens Updated Annotation Release 109.20201120 on GRCh38.p13

B

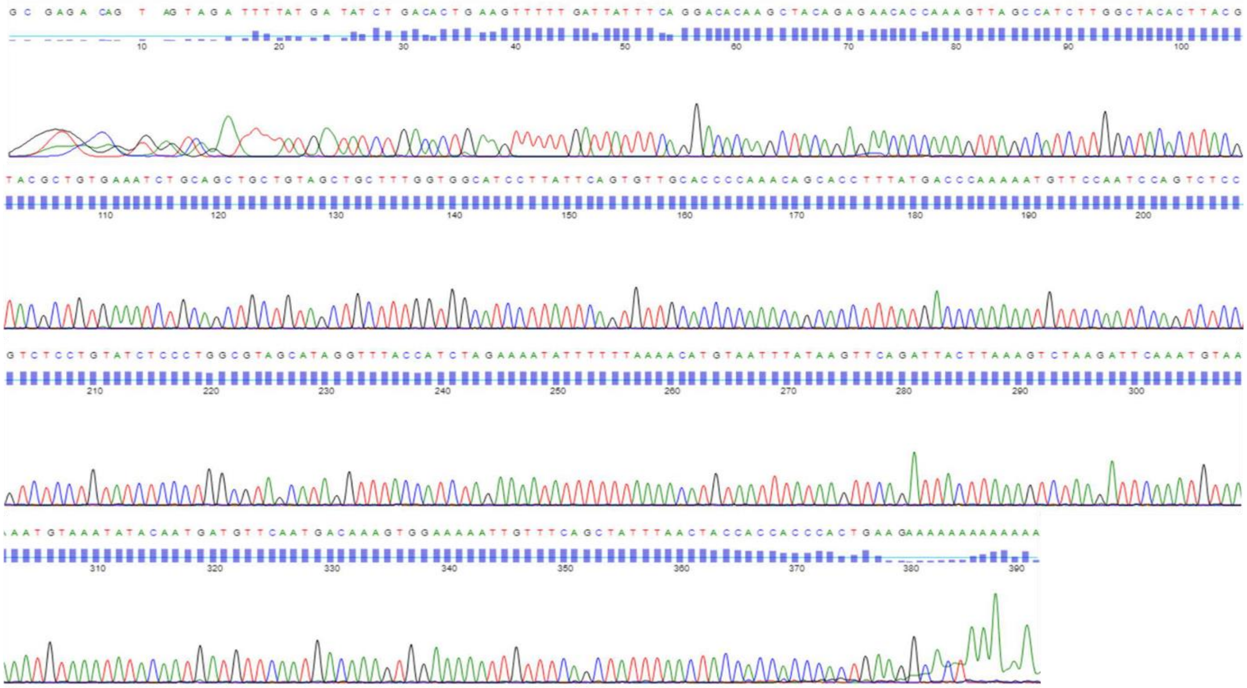

C

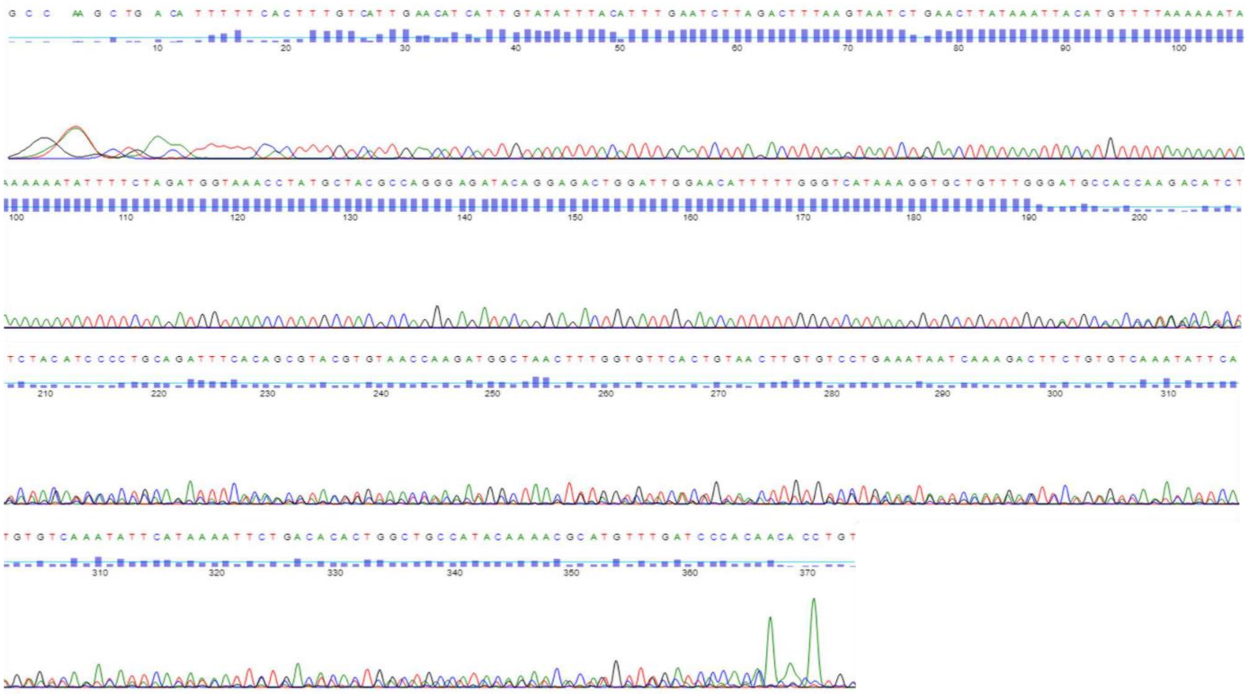

Supplemental Figure S3: Sanger sequencing confirmed successful knockout of STRAP in SK-N-BE(2) cells. (A) The same approach was utilized in analyzing the Sanger sequencing results of SK-N-BE(2) (BE) WT and BE STRAP KO DNA as previously described in Supplemental Fig. 2. BE STRAP KO demonstrated a deletion (~20 bp) that corresponded to the location of the STRAP guide RNA (gRNA). The designed gRNA and the STRAP primer pair (forward and reverse primers) were also aligned and their position is shown in relation to the sequencing results. (B, C) Chromatograms of BE WT (C) and BE STRAP KO (D) resulting from Sanger sequencing.

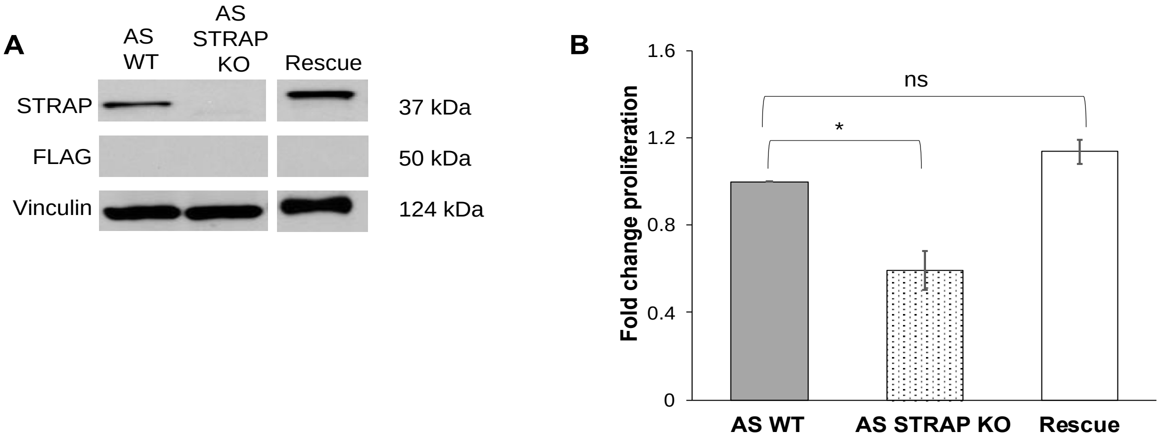

Supplemental Figure S4: Re-introduction of STRAP DNA rescued the malignant phenotype in STRAP knockout cells. (A) AS STRAP KO cells were plated and

transiently transfected for 72 hours with FuGENE® plus STRAP plasmid (Rescue). Immunoblotting confirmed both the absence and subsequent rescue of STRAP protein expression in the AS STRAP KO cells as well as FLAG expression in the rescue cells, confirming the presence of the STRAP plasmid. (B) To examine the phenotype of the STRAP rescue cells, proliferation was investigated using CellTiter 96® assay. AS STRAP KO led to a decrease in proliferation as previously noted. When STRAP was re-introduced into the AS STRAP KO cells, proliferation returned to the baseline level of the AS WT cells. Data reported as mean fold change proliferation ± SEM. Experiments were repeated with at least three biologic replicates. *p≤0.05, ns: non-significant.

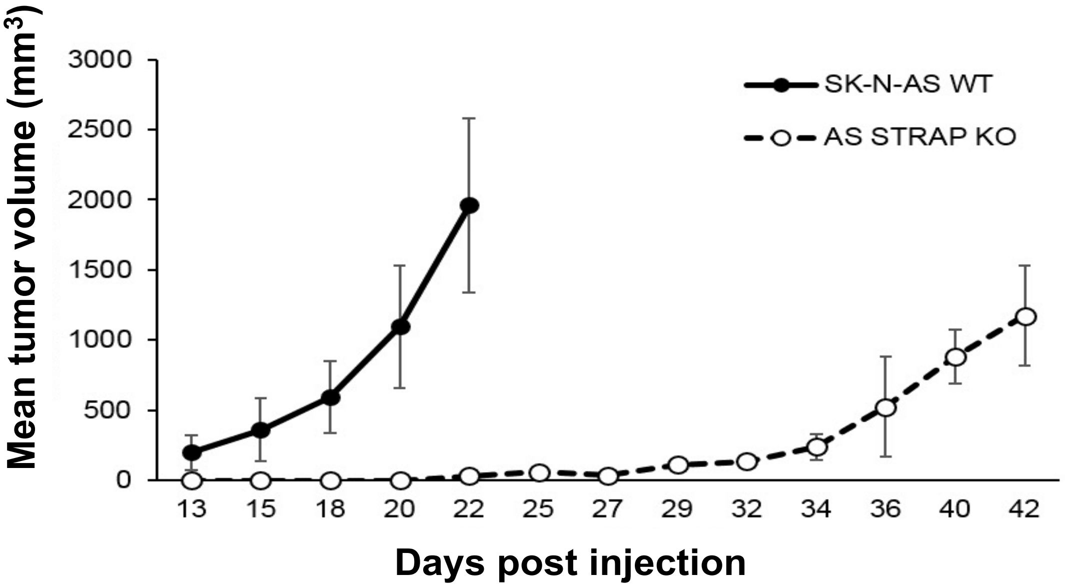

Supplemental Figure S5: STRAP knockout decreased neuroblastoma tumor growth in vivo. AS WT or AS STRAP KO cells (1.8 × 106) were injected into the flanks of athymic nude mice (n=9 per group). Tumor volumes were measured three times a week, and tumors were harvested when IACUC parameters were met. STRAP KO tumors had a significantly decreased mean tumor volume over time compared to WT tumors. Results are reported as mean tumor volume with the formula [(width2 × length)/2] mm3 with width being the smallest measurement ± SEM.

	AS WT	AS STRAP KO
Mean % G1 (± SEM)	53.78 (± 5.01)	62.70 (± 0.83)
Mean % S (± SEM)	28.23 (± 3.07)	17.53 (± 2.46)
Mean % G2 (± SEM)	13.30 (± 1.79)	12.72 (± 1.39)

Supplemental Table S1: STRAP knockout diminished progression through the cell cycle. AS WT and AS STRAP KO cells were serum starved for 24 hours then plated in media and stained with propidium iodide after 24 hours. Flow cytometry was used

to analyze progression through the cell cycle. AS STRAP KO had significantly decreased percentage of cells in the S phase and an associated increased percentage of cells in the G1 phase. Data are shown in tabular form with values representing mean percentage of cells in phase from three independent biologic experiments (± SEM). *p≤0.05**.**

A

Limiting dilution data entered.

Counter	Dose	Tested	Response	Group
1	1000	36	36	AS_WT
2	500	36	36	AS_WT
3	100	36	36	AS_WT
4	50	36	35	AS_WT
5	50	36	31	AS_WT
6	20	36	22	AS_WT
7	10	36	2	AS_WT
8	1000	36	28	AS_STRAP_KO
9	500	36	20	AS_STRAP_KO
10	100	36	2	AS_STRAP_KO
11	50	36	0	AS_STRAP_KO
12	50	36	0	AS_STRAP_KO
13	20	36	0	AS_STRAP_KO
14	10	36	0	AS_STRAP_KO

B

Confidence intervals for 1/(stem cell frequency)

Group	Lower	Estimate	Upper
AS_STRAP_KO	1053.7	796.0	601.3
AS_WT	29.9	24.2	19.6

C

Overall test for differences in stem cell frequencies between any of the groups

Chisq	DF	P.value
403	1	1.21e-89

Supplemental Table 2S: STRAP knockout decreased neuroblastoma tumorsphere formation. (A) An extreme limiting dilution analysis was utilized to assess tumorsphere formation. Experiments were repeated with at least three biologic replicates. Raw data entered into the analysis tool corresponding to Fig. 5 C is shown. Cells were plated in conditioned media in non-adherent conditions at decreasing cell concentrations per well (1000 to 10, dose). Wells with tumorspheres present were counted (response) in relation to the total number of wells (tested). A plot of the log proportion of negative cultures vs. the number of cells plated is shown in Fig. 5 C, with the slope of the line representing the estimated log-active stem cell fraction. (B) Estimated and 95% confidence intervals (with both lower and upper bounds reported) for the 1/(stem cell frequency) for each group. AS STRAP KO cells had a higher number of cells required for sphere formation, indicating decreased ability to form tumorspheres, compared to AS WT cells. (C) Table showing the test for differences in stem cell frequencies between the two groups.
